# The Plans and Purposes of the Johns Hopkins Hospital

**Published:** 1889-08-24

**Authors:** John S. Billings

**Affiliations:** Surgeon, U.S. Army


### The Plans and Purposes of the Johns Hopkins Hospital.*

By John S. Billings, M.D., Surgeon U. S. Army.

( Continued from page 315 )

### II.—CARDINAL PRINCIPLES.

The last point to which I shall refer, which was kept in view by the trustees in deciding upon the plans, was the general appearance of the buildings and grounds.

Mr. Hopkins gave no specific directions as to the buildings, but he directed that the grounds should be properly enclosed by iron railings, and so laid out and planted as to be a solace to the sick and an ornament to the city, and it was evident that the buildings should be of the same character so far as their purpose would admit. It was therefore decided that, while no utility should be sacrificed for the sake of architectural ornament, and the main purpose which I have referred to should be fully worked out in the plans before any attention was paid to external appearance, it was fit and proper that the buildings should form an ornament to the city, and a suitable monument to the memory of the donor.

Bearing in mind, then, these main principles, to provide for the proper care of the sick, both rich and poor, to provide for the highest class of medical education, to increase and diffuse knowledge, to provide trained nurses for both hospital and city, to provide a dispensary, and to make the buildings and grounds ornamental and attractive, let us see how the problem has been thus far worked out.

I will begin with the arrangements for securing that article of prime necessity in a hospital—viz., pure air. Air supply and ventilation in this climate are inseparably connected with heating for a considerable portion of the year, for comfortable warmth must be secured, and on the means of doing this must largely depend the methods of ventilation and their success. The temperature of Baltimore may vary from 103deg. in the shade to 17deg. below zero, F., hence its perfect hospital must be one which would answer for the tropics or for Northern Russia. To secure this, double walls, with air spaces, were given to the buildings, and a system of heating by the circulation of hot water was adopted for the wards. This system consists of central boilers, from which flow and return pipes extend beneath every building, connected with heating coils, of which there is one for every two beds in the ward above. The temperature in these coils can be exactly regulated to any temperature between 150deg. F. and the temperature of the external air by simply regulating the velocity of the flow of water by the valves attached to each coil, and thus it is quite possible to give one pair of beds a temperature of 70deg. and another pair in the same room, at a little distance, a temperature of 60deg. F., to suit the needs of different cases. The 80,000 gallons of water contained in this heating apparatus go round and round, carrying heat from the furnaces to the wards, but every building has it own independent means of ventilation, and it is not possible to go from one ward into another without going into the open air on the way, so that foul air, if any forms, cannot spread from one building to another. Nevertheless, the buildings are so connected by corridors and underground tunnels that in passing from one to another there is no exposure to rain or snow, and the least possible to cold air, while the food is not exposed at all. This is not the place to describe the ventilation. I will only call your attention to the fact that the temperature of the incoming air by any bed is easily changed by turning a valve, while the quantity of air is not changed; to the arrangement for taking foul air from either the bottom or top of the ward, or from both, and to the fact that all this has been thoroughly tested during two winters, and found to give the results hoped for.

One of the peculiarities of the wards is that all the service-rooms are collected at the north end, leaving the south end free of obstruction and fully exposed to the sun, the end of the ward being a large bay window looking out on the central garden, and with a floor which can be warmed so that the patients able to sit there can be thoroughly comfortable. Another peculiarity of the sick wards is the arrangements for easy cleansing, and to prevent possible accumulations of dust in corners and crevices. Corners are to a great extent done away with, and easy curves given in their place; even at the junction of the floor and walls there is a curve instead of the usual right angle, and I advise you to look at it and see how it has been produced, for it ought to become fashionable, and take the place of the old mop-board in all well-constructed houses. So, also, the doors have not the usual moulding about the panels, giving recesses which is almost impossible to clean.

One of the wards is especially arranged for cases which may be either contagious or offensive. In this building each patient is in a room by himself, and all these rooms open into a corridor through which the wind is always blowing. There are many details about this isolating ward which are worth looking at, but which I have not time now to refer to, and I must omit details about the pay ward, the octagon ward, and the peculiar fittings and conveniences of the kitchen, laundry, apothecaries' building, etc., for the same reason.

Let us pass now to the second object of the hospital—the giving means for higher medical education, and see what has been done for that. In the first place, there is a large amphitheatre with appended rooms for the reception of accidents and emergencies of all kinds. In the second place, provision is made for at least thirty students to reside constantly in the hospital, and devote themselves under proper guidance to the study of disease and the practical care of the sick. It is intended that these places shall be open only to those who have had a thorough previous training, and who have shown themselves to be fitted to undertake this important part of their studies. As a rule, not more than 5 per cent. of medical graduates have had any opportunities worth speaking of to study and treat diseases in the living man when they receive their diplomas. They have to get this experience on their first patients, and sometimes the experience is rather hard—for both doctor and patient. This hospital has provided for the class of the medical school in the last year of their studies good rooms, with bathrooms, a dining-room, and other conveniences, and here they can be taught the actual daily work of a physician, for which all their previous studies are only preparatory. Many of the arrangements of the hospital have been made with reference to this instruction. It is a great laboratory for teaching the practical applications of the laws of hygiene to heating, ventilation, house drainage, and other sanitary matters. All pipes and traps are either exposed to view, or can be seen by merely opening a door, and in the tunnel beneath the corridor you can study at your leisure the complicated and yet simple arrangement of pipes for gas, steam, water, sewage, etc., which are usually buried and remain a profound mystery to everyone except the plumber, and often puzzle even him.

Closely connected with this subject of teaching is that of increasing our knowledge of the causes, symptoms, results, and treatment of disease; in fact, one cannot be

* An address delivered at the opening of the hospital, May 7, 1889.

thoroughly and well done without the other, and hence many of the provisions for the one are also useful for the other. For example, to go back to our system of heating and ventilation, there are many points connected with it which are destined for experimental work, to compare steam with hot-water heating, to determine the velocity of water at different temperatures, to compare ventilation by aspiration with that by propulsion, or by upward currents with those drawn downward.

One structure is very largely devoted to and fitted for experimental research, and that is the pathological laboratory, where the causes, processes, and results of disease are to be studied. Upon the results obtained in that laboratory may yet depend the saving of many lives, the relief of unspeakable agony, the warding off of pestilence from the city, and, to put it in a strictly business light, the value of real estate and the rate of taxation of this community. We are on the verge of great advances in our knowledge of the causes and methods of disease, and I feel sure that these will be only preliminary steps to far greater and better knowledge of how to prevent or to treat them than we now have. The probable length of life of the new-born infant to-day is not much more than half what it ought to be; the practical productive period of the life of our men and women is shortened and interrupted by unnecessary disease and suffering; but, remember, if these things are to be amended, it is not merely by teaching old doctrines; we must open fresh windows and let in more light, so that we can see what these obstacles really are. It is in this work of discovery that it is hoped that this hospital will join hands with the university, and it is in this hope that some of the structures around you have been planned and provided.

*(To be continued.*

